# The Function of Color and Structure Based on EEG Features in Landscape Recognition

**DOI:** 10.3390/ijerph18094866

**Published:** 2021-05-03

**Authors:** Yuting Wang, Shujian Wang, Ming Xu

**Affiliations:** 1 Henan Key Laboratory of Earth System Observation and Modeling, Henan University, Kaifeng 475004, China; wyt2019@vip.henu.edu.cn (Y.W.); 104752190013@henu.edu.cn (S.W.); 2 College of Environment and Planning, Henan University, Kaifeng 475004, China

**Keywords:** color, structure, landscape recognition, electroencephalography (EEG)

## Abstract

Both color and structure make important contributions to human visual perception, as well as the evaluation of landscape quality and landscape aesthetics. The EEG equipment liveamp32 was used to record the EEG signals of humans when viewing landscape images, structure images with filtered color, and color images with a filtered structure. The results show that the SVM classifier was the most suitable classifier for landscape classification based on EEG features. The classification accuracy of the landscape picture recognition was up to 98.3% when using beta waves, while the accuracy of the color recognition was 97.5%, and that of the structure recognition was 93.9% when using gamma waves. Secondly, color and structure played a major role in determining the alpha and gamma wave responses, respectively, for all the landscape types, including forest, desert, and water. Furthermore, structure only played a decisive role in forest, while color played a major role in desert and water when using beta waves. Lastly, statistically significant differences between landscape groups and scenario groups with regard to alpha, beta, and gamma rhythms in brain waves were confirmed. The reasonable usage and layout of structure and color will have a very important guiding value for landscape aesthetics in future landscape design and landscape planning.

## 1. Introduction

The measurement of brain activity is an objective way of assessing the physiological perception of engagement with the landscape, environment, or other objects [1,2,3]. Brain imaging is helpful to measure the effects of unconscious stimuli [4,5]. Research has discussed the interplay between landscape types and the physiological response of human beings. Recent laboratory-based neuroimaging studies indicated that various environments may be associated with characteristic patterns of brain activity [6,7]. EEG frequency features have commonly been used in EEG signals. Generally, high-amplitude signals in the low-frequency range are observed when the subjects are in a calm state, whereas high-amplitude signals in the high-frequency range are obvious in a stimulated state [8,9]. EEG features (frequency-domain features, time-domain features, and spatial-domain features) in EEG signals represent the brain region activities. Thus far, there have been many studies using EEG technology and machine learning for recognition with good classification accuracy, e.g., emotion recognition [10,11,12,13], object structure recognition [14,15], color recognition [16], and landscape and animal image recognition [17]. Some studies have gradually applied EEG technology to different fields, including environmental perception and landscape assessment [6,18,19,20], while others have also explored the impact of specific environmental characteristics on the natural environment [21,22,23].

Color is a basic aspect of human perception. It is known that color also affects human spirit and emotion. Moods and behaviors can be changed by colors [24]. Color signals improved the survival of early humans [25,26]. Differences in human color perception according to physical and psychological experiments have been discussed [27]. British color planning expert Lancaster proposed the concept of “color landscape”. Expressing that a localized or personalized landscape can be achieved by controlling the color of elements in the environment [28]. On the other hand, object recognition has revealed the role of structure information in higher-level vision [29,30]. Data from behavioral studies and neuropsychological studies have suggested that color (as surface features) contributes to structural object recognition [31,32]. At the same time, color information plays an important role in the visual recognition of natural objects [33].

The quantitative analysis of color is important for a scientific evaluation of landscape aesthetic quality and forest landscape management [34]. Landscape type also plays a decisive role in the visual aesthetic quality of the local landscape. The complexity of landscape structure will affect people’s evaluation of landscape aesthetic quality to a certain extent. For example, the structure of the forest community and the spatial distribution of population affect the landscape aesthetic quality [34,35].

In this paper, we aimed to analyze the role of color and structure in landscape recognition by using the objective quantitative index of EEG features. We mainly focused on color and structure because they play important roles in landscape evaluation and recognition, as well as affect people’s spirit and emotion. EEG signals have been not only used as a tool to supplement surveys or expert opinion commonly utilized in the landscape evaluation field [19], but also supported by functional magnetic resonance imaging (fMRI) [36] and near-infrared studies [37,38]. Therefore, research on the role of color and structure as a function of EEG features in landscape recognition can provide a necessary basis for landscape perception and landscape recognition in landscape evaluation. This study will provide a method and guidance for use in summarizing the weight of color and structure in landscape classification.

Many previous studies have shown that color and structure play an important role in environmental assessment and landscape recognition, especially in human perception of the environment or landscape [21,22,23,25,26,27,34,35]. However, the influence of color or structure on landscape recognition is not clear as a function of the objective index of EEG features. Three hypotheses are proposed to explore the role of color and structure in landscape recognition:

(1). The recognition accuracy of different colors, structures, and landscape types varies, whereas the classification accuracy for different scenarios in different landscapes is similar;
(2). Color and structure play different roles in different landscape types;
(3). The distribution of brain regions is different in different scenarios and landscape types.

## 2. Materials and Methods

**Figure 1:**
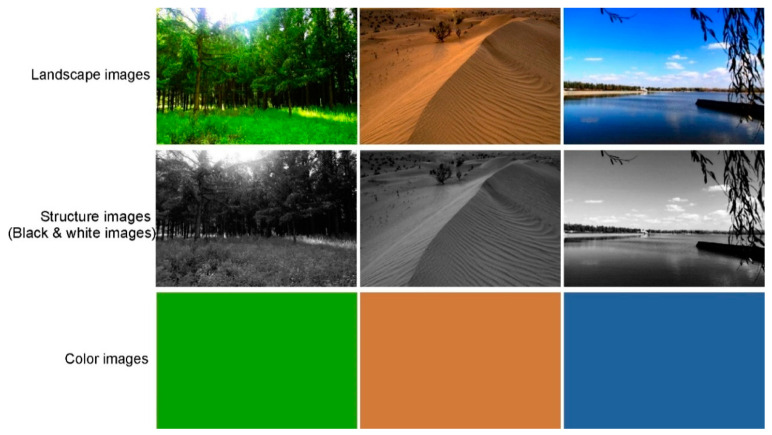
Examples of experimental images.

We selected the experimental images (Figure 1) for this study through consultation with several experts.

### 2.1. Materials

Firstly, five photographs were selected to represent each type of common natural setting, including forest, desert, and water. Then, 10 people with more than 5 years of experience in landscape design were asked to rate each photo on a scale of 1–5. The highest-scoring photographs were chosen according to the cumulative scores. The landscape images were color-filtered to form structure landscape images (i.e., black and white landscape images) and then structure-filtered to form corresponding color images. Accordingly, we obtained three groups of images (landscape images, structure images, and color images) for this experiment. In Adobe Photoshop CS4, the color-finder tool takes an RGB value of 10 points in the landscape images and takes the average value. This process was repeated three times to get the average RGB value; then, the color images were quarried. The structured images were grayscale-converted from landscape images to black and white using Adobe Photoshop CS4 with the default RGB values (best for color comparison) and no special retention of brightness or shadow. Secondly, each participant’s EEG signals were collected and evaluated. Lastly, EEG results were analyzed to assess the perception of participants in different scenarios (landscape images, structure images, and color images) and different landscape types (forest, desert, and water). The role of color and structure in landscape recognition was concluded on the basis of EEG features.

In the laboratory, the room was completely closed and without noise. A 15 inch display device was put in front of the subjects. There were nine different images, each of which remained on screen for about 20 s (20 repeats for each image, with 2 s of stimulation each). After a series of images, the volunteers had a rest.

### 2.2. Subjects

A total of 20 volunteers aged between 25 and 55, nine women and 11 men, participated in the experimental procedure. The participants had varied employment situations, such as university staff, social workers of various industries, and university graduates. We described the purpose of the experiment before beginning, and participants were asked to be right-handed, with no color blindness, and in good physical health, without any history of mental disorder. If they met the requirements and agreed to continue the experiment, they then signed an informed consent form before testing. The study was approved by the school’s ethics committee.

### 2.3. Method

**Figure 2:**
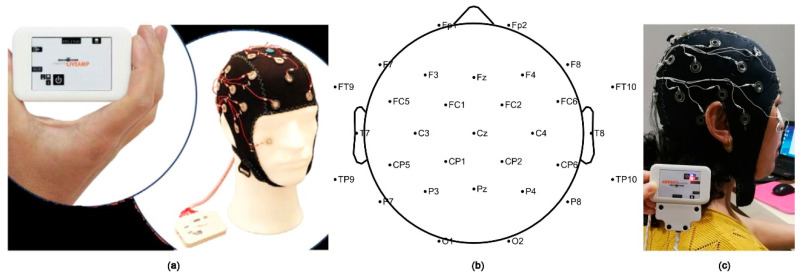
(**a**) Brain Products (LiveAmp) device with 32 channels used in the experiment; (**b**) electrode distribution in brain region; (**c**) example of a volunteer.

In this study, the Active System produced by Brain Products (LiveAmp, Brain Products GmbH, Gilching, Germany) with 32 channels was used to obtain the signals of the brain activity (Figure 2). The original EEG data were analyzed by the EEGlab, which is a toolbox for processing continuous EEG signals. The EEG signals were detrended using the average of left and right mastoids as a reference. Then, eye electrical, electromyography, electrocardiography, power frequency interference, and other disturbance artefacts were removed by independent component analysis (ICA) [39,40,41]. Next, the EEG signals were segmented into contiguous 2 s windows, and any segments which retained artefacts were rejected [42]. The experiment used the international 10–20 system and a 32-channel electrode cap. Therefore, a total of 1800 (9 × 20 × 10) data samples were generated. Then, 250 Hz downsampling and 0.5–50 Hz filtering were performed to obtain the preprocessed EEG datasets. Fast Fourier transform was used to extract frequency band information. The frequency-domain features were extracted to obtain the logarithmic frequency energy values of the waves in five frequency bands: delta (1–4 Hz), theta (4–8 Hz), alpha (8–13 Hz), beta (13–30 Hz), and gamma (>30 Hz) [43].

The absolute EEG value for various types of pictures show that the natural landscape play a certain role in recovery from stress, whereby alpha waves (8–13 Hz) and beta waves (13–30 Hz) are the most suitable indicators [3]. Gamma waves (>30 Hz) are associated with meditation and happiness [44], as well as high-level information processing [45,46]. Another study also found a correlation between relaxation therapy and alpha waves [21]. As delta and theta waves are mainly presented during sleep, alpha, beta, and gamma waves were mainly used in this study for different landscapes and scenarios.

The EEG data were randomly divided into training (70%) and test (30%) data. For our purposes, we used 10-fold cross-validation to train and test extracted features for all classifiers. For KNN, we used k = 5 as a baseline for comparison with other classifiers. For random forest, we used a total of 500 trees. We used LIBSVM software [47] to implement the SVM classifier and employ the linear kernel.

### 2.4. Statistical Analysis

Firstly, machine learning (K-nearest neighbor, random forest classifier, and support vector machine) was used to classify different landscape types, colors, and structures as a function of brain waves after the pretreatment of EEG signals; then, the recognition accuracy in different situations was obtained. Secondly, the relative weights of structure and color in landscape recognition were calculated, and the method approximated the average increase in *R*^2^ upon adding predictive variables to all possible submodules [48,49,50], followed by using the reweights function in R software [51]. Lastly, variance analysis was used to test differences between groups with the same sample size. The Tukey HSD test was carried out for a binary comparison between different scenarios and different landscape types. The significant differences in different scenarios and using different landscape types were identified. All the statistical analyses were performed using R and Matlab. Graphs were completed in Matlab and Excel, and graph combination was completed in CorelDRAW2018.

## 3. Results

### 3.1. Recognition Accuracy of Landscape, Structure, and Color Based on Brain Waves

**Figure 3:**
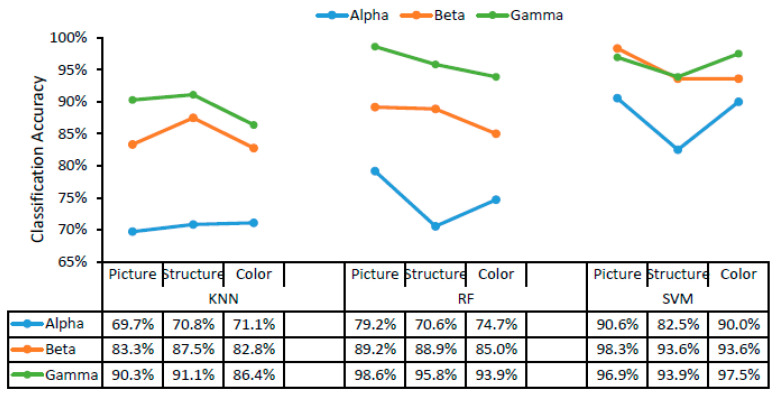
The accuracy of color, structure, and landscape image classification as a function of brain waves. KNN, K-nearest neighbor; RF, random forest; SVM, support vector machine.

The classification accuracy of the three different classifiers for landscape, structure, and color are displayed in Figure 3 as a function of alpha, beta, and gamma waves. SVM was the classifier with the highest recognition accuracy for colors, structures, and landscapes using alpha, beta, and gamma waves, followed by RF and KNN (Figure 3). Using the SVM classifier, the trend of recognition accuracy was gamma > beta > alpha, while similar trends were confirmed using both the RF classifier and the KNN classifier. The accuracy of landscape image classification was higher than that of structure and color image classification using alpha, beta, and gamma waves in RF and SVM. Using the SVM classifier, the accuracy of landscape image recognition was up to 98.3% using beta waves, while the recognition accuracy of colors corresponding to the landscape was 97.5% using gamma waves, and that of structures was 93.9% using gamma waves. Using the RF classifier, the accuracies of landscape, structure, and color were 98.6%, 95.8%, and 93.9%, respectively, using gamma waves.

**Figure 4:**
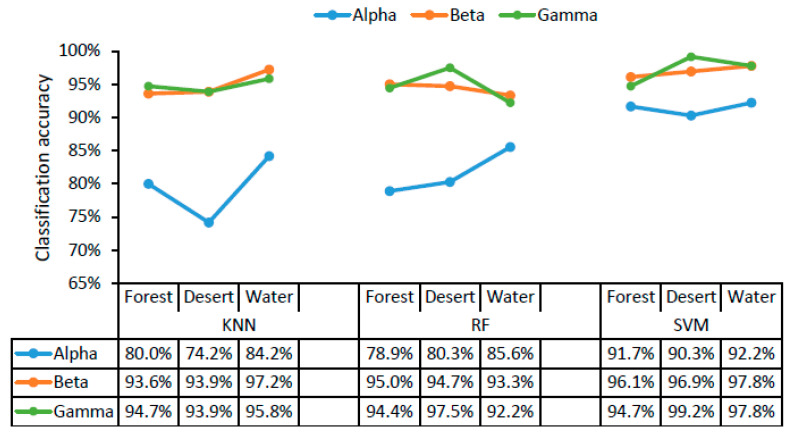
The accuracy of classifying landscape types (forest, desert, and water). KNN, K-nearest neighbor; RF, random forest; SVM, support vector machine.

The classification accuracy of the three different classifiers for three different scenarios (forest, desert, and water landscapes) are displayed in Figure 4 as a function of alpha, beta, and gamma rhythms in brain waves. The classifier with the highest recognition accuracy for landscape color images (with structure filtered out), landscape structure images (with color filtered out), and raw landscape images was SVM using alpha, beta, and gamma waves in all cases (Figure 4). Using the SVM classifier, the accuracy of different scenarios was higher, exceeding 90% for alpha, beta, and gamma waves with all landscape types. The classification accuracy for the three different scenarios was 91.7–92.2% using alpha waves, 96.1–97.8% using beta waves, and 94.7–99.2% using gamma waves. There was little difference in classification accuracy across the three landscape types. The high recognition accuracy for the different scenarios indicated that color and structure both play an important role in landscape recognition.

According to the results in Figure 3 and Figure 4, SVM was the most suitable classifier in this study, with accuracies exceeding 82.5% in all cases, which were relatively higher than those obtained using KNN and RF with alpha, beta, and gamma waves. In addition, the goodness of fit, specificity, and sensitivity of different classifiers for different landscape types and different scenarios (Appendix A) were calculated when choosing the most suitable classifier.

### 3.2. Weight and Brain Distribution of Structure and Color in Landscape Recognition

**Figure 5:**
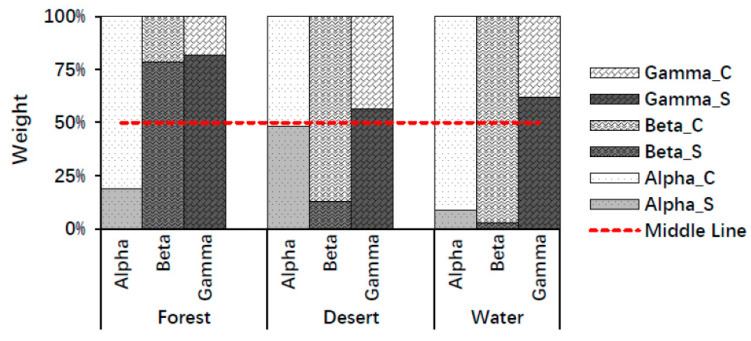
The weights of structure and color in landscape image recognition using mean values of the whole brain. Alpha_S, Beta_S, and Gamma_S indicate structure weights using alpha, beta, and gamma waves; Alpha_C, Beta_C, and Gamma_C indicate color weights using alpha, beta, gamma waves.

According to the average values of the whole brain, color played a major role in stimulating alpha waves, whereas structure played a major role in stimulating gamma waves for all landscape types (forest, desert, and water). In addition, structure played a major role in forest recognition, whereas color played a major role in desert and water recognition using beta waves. Here, a major role was defined as a weight in landscape recognition greater than 50% (Figure 5). The above findings may be due to the structure of the forest being the most complex among the three landscapes, while the structure of other landscapes is relatively simple.

**Figure 6:**
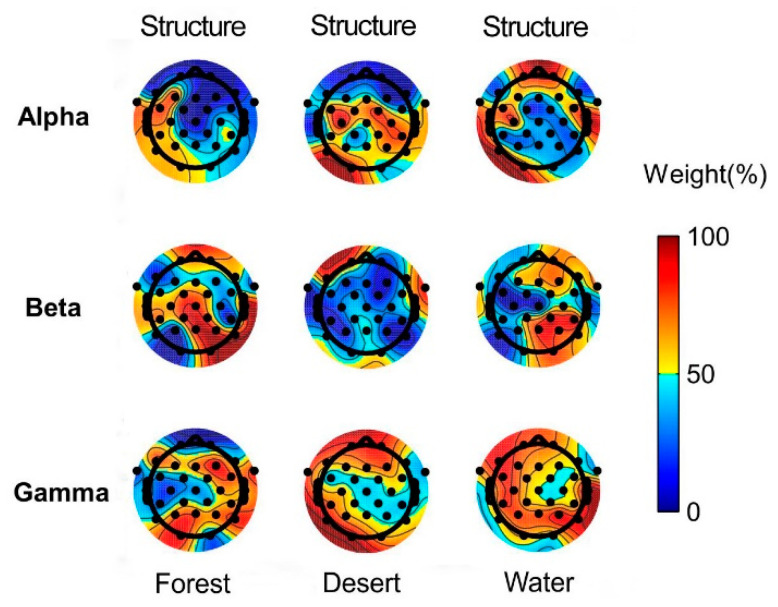
The weights of structure and color in landscape image recognition for different landscape types using alpha, beta, and gamma waves. Predominantly orange electrodes represent the dominance of structure, whereas predominantly blue electrodes represent the dominance of color in different landscapes.

The weights of structure and color in landscape image recognition using alpha, beta, and gamma rhythms in brain waves of the whole brain are presented in Figure 6.

### 3.3. Analysis of Different Landscapes and Scenarios Based on EEG Features

Table 1 shows the results of two-way ANOVA, suggesting a significant difference in the human perception of scenario and landscape type. The group differences according to scenario were found to be significant for alpha, beta, and gamma waves (*p* < 0.01), while the differences according to landscape type were also significant for beta waves (*p* < 0.00). However, differences according to landscape type were not significant for alpha and gamma waves (*p* = 0.26 and 0.55, respectively). Moreover, the interaction effect between scenario and landscape type was significant. Since the interaction effect between two factors was verified by the above analysis, we performed a Tukey HSD test to determine which electrode underlying each factor affects human perception.

**Figure 7:**
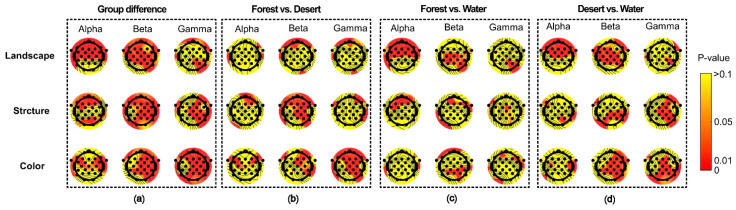
The difference in distribution of brain waves for alpha and beta waves among landscapes in different scenarios. (**a**) the group differences among different scenarios, (**b**) the difference between forest and desert, (**c**) the difference between forest and water, (**d**) the difference between desert and water.

For different scenarios, there were significant differences among landscape groups, structure groups, and color groups, which were reflected in multiple electrodes of the whole brain (Figure 7a). Furthermore, differences between each group were also reflected (Figure 7b–d). A significant difference between forest and desert was mainly reflected in most electrodes for beta waves in landscape and structure, as well as for beta and gamma waves in color (Figure 7b). A significant difference between forest and water was mainly shown for alpha waves in landscape and structure, as well as for beta waves in color (Figure 7c). A significant difference between desert and water was mainly displayed for alpha and beta waves in landscape, as well as for beta and gamma waves in structure and color (Figure 7d).

**Figure 8:**
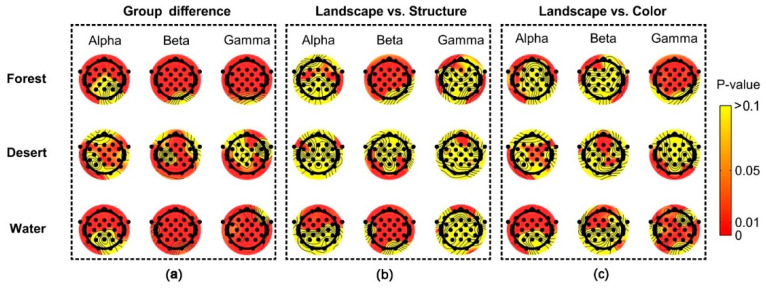
The difference in distribution of brain waves among scenarios in different landscape types. Landscape indicates landscape images, Structure indicates structure images, and Color indicates color images. (**a**) the group differences among different landscape types, (**b**) the difference between landscape images and structure images, (**c**) the difference between landscape images and color images.

Significant group differences among scenarios (landscape images, structure images, and color images) were reflected for most electrodes using alpha, beta, and gamma rhythms in brain waves (Figure 8a). Significant differences between landscape and structure images were mainly shown for most electrodes using beta waves in forest and desert, as well as alpha and beta waves in water (Figure 8b). Significant difference between landscape and color images were mainly shown using gamma waves in forest, beta waves in desert, and alpha and gamma waves in water (Figure 8c).

## 4. Discussion

### 4.1. Recognition Accuracy of Landscape, Structure, and Color Based on Machine Learning

The recognition of landscape images, structure images, and color images, as well as the recognition of different scenarios, showed that SVM was the classifier with the highest accuracy among the three landscape types (Figure 3 and Figure 4). The classification accuracy was 98.3% for landscape types using beta waves, 97.5% for colors (green, blue, and orange) using beta waves, and 93.9% for structures using gamma waves. Rasheed et al. used linear, polynomial, and radial basis function kernels in a support vector machine to classify red, green, and blue colors on the basis of EEG signals, yielding accuracies of 84%, 89%, and 98%, respectively [16], similar to the results for color classification accuracy in this study (97.5%), thus indicating their reliability. Khasnobish et al. classified objects on the basis of EEG signals using 10 objects of various regular geometrical shapes (cone, cube, sphere, hemisphere, cylinder, prism, and hexagonal base cylinder) and two irregularly shaped objects (lock and mouse). The recognition accuracy was 88.34% for pure tactile, 81.1% for pure visual, and 82.2% for a mixture of tactile and visual elements. The average classification accuracy over all three object exploration modalities was 83.89% [14]. Rus et al. found that the EEG features in gamma wave were more suitable for object recognition, and they used three different classifiers (SVM, KNN, and ANN) to classify objects, yielding accuracies of 89.5%, 89.5%, and 83%, respectively [15], consistent with the highest classification accuracy obtained using gamma waves in all brain waves in this study. Using EEG signals, Lam et al. implemented a single-layer neural network method to identify and classify landscape images and animal images, yielding an average accuracy of 91.15%, in which the average recognition rate of landscape images was 89.69%, and that of animal images was 92.34% [17]. Our classification accuracy results of 98.3% using beta waves, 96.9% using gamma waves, and 90.6% using alpha waves for landscape images with an SVM classifier are a little higher than Lam et al.’s result of 89.69%.

### 4.2. Role of Color and Structure in Landscape Identification

According to the average values of the whole brain, color plays a major role in stimulating alpha waves, whereas structure plays a major role in stimulating gamma waves for all landscape types (forest, desert, and water). In addition, structure played a major role in forest recognition, while color played a major role in desert and water recognition using beta waves. A major role denotes a weight in landscape recognition greater than 50% (Figure 5). The locations of the brain where structure played a major role were not the same as those identified for colors using alpha, beta, and gamma waves in different landscape types (Figure 6). The reason for this finding may be due to the structure of forests being the most complex among the three landscapes, while the structure of the other landscapes is relatively simple and homogeneous. Alpha waves normally play a major role in the relaxed state [21,52]. Our finding that color plays a major role in stimulating alpha waves for all landscapes suggests that color is very important in recognizing natural landscapes that induce relaxation and stress recovery. Gamma waves are associated with high-level information processing and a high cognitive state [45,46,53,54,55]. Our finding that structure plays a major role in stimulating gamma waves for all landscape types is consistent with the above conclusion. Beta waves play a major role when people are alert or stimulated [3]. Among the three landscape types, the structure of forests is relatively complex, while the structure of deserts and water is relatively simple. Consequently, when people were stimulated by the corresponding landscape, structure played a major role in the recognition of forest landscape, while color played a major role in the recognition of desert and water using beta waves. Another possible reason may be closely related to the plant diversity of the forest, where the tree density level is more conducive to a sense of relaxation. Previous studies have shown that moderate vegetation levels are associated with optimal physiological outcomes [56]. The structure of the forest landscape in this study was relatively complex with a medium vegetation level. It can be concluded that, in simple landscapes, color plays a very important role in influencing people’s perception, whereas, in relatively complex landscapes with a high species diversity, structure plays a major role. In summary, both color and structure are important for landscape recognition. Therefore, their reasonable usage and layout can be very important for guiding landscape aesthetics in future landscape design and landscape planning.

Compared with other BCI-based studies [18,19,23,26,36,57,58], the electrode distribution in this study was denser. Thus, relatively more detailed brain distribution differences and higher spatial brain resolution were obtained. In order to clearly reflect the role of structure and color, we also examined the differences between each group. Our finding confirmed a statistical significance in landscape and scenario recognition using alpha, beta, and gamma waves, thereby revealing that structure and color are important for landscape recognition as their absence led to significantly differences.

Future research should further explore landscape structures and colors that can cause an increase in the alpha rhythm of brain waves (inducing a physiological relaxation and stress recovery state), which can greatly help in the identification of landscapes that provide spiritual cultural services for human beings. Moreover, there is a need for further research into the relationship between the time of human exposure to the natural landscape and the process of brain waves of humans. Future research should focus on the differences in the individual perception of landscape physiology, including an increase in sample size and age range and more varied employment situations, as well as differences in gender, education level, residence, property income, and other social attributes. It may be helpful to find relevant patterns between psychophysiological trends and individual social characteristics.

## 5. Conclusions

With the development of EEG technology in recent years, direct evidence of human brain activity has enabled new pathways for landscape research. The observation of brain activity can provide the possibility of identifying mechanisms underlying perceptual reactions associated with environmental stimuli.

This study showed that the SVM classifier was the most suitable classifier for landscape classification based on EEG features. Secondly, color played a major role in stimulating alpha waves, while structure played a major role in stimulating gamma waves for all the landscape types (forest, desert, and water). In addition, structure played a major role in forest recognition, while color played a major role in desert and water recognition using beta waves. Lastly, a statistical significance difference in landscape and scenario recognition using alpha, beta, and gamma rhythms in brain waves was confirmed.

In conclusion, the leading role of structure or color in landscape recognition is not always certain; thus, a reasonable usage and layout of structure and color can be a very important guiding value for landscape aesthetics in future landscape design and landscape planning. This could have constructive significance for the development of beautiful countries and cities. Furthermore, the significant difference in the stimulation of brain waves according to landscapes highlights their influence on people’s perception, which is a great reference value for the rational planning of multifunctional landscapes with both recreational and educational function; it also provides a method for the quantification of people’s spiritual value of cultural ecosystem services.

## Figures and Tables

**Table 1:** Results of ANOVA.

Factor	Df	SumSq	MeanSq	F_value	Pr (>F)
Alpha wave					
Scenario	2	0.31	0.15	4.67	0.01 *
Landscape	2	0.09	0.04	1.35	0.26
Scenario: Landscape	4	1.41	0.35	10.70	0.00 ***
Residuals	171	5.62	0.03		
Beta wave					
Scenario	2	0.16	0.08	11.54	0.00 ***
Landscape	2	0.12	0.06	8.47	0.00 ***
Scenario: Landscape	4	0.06	0.01	2.09	0.08 ^•^
Residuals	171	1.16	0.01		
Gamma wave					
Scenario	2	0.34	0.17	15.42	0.00 ***
Landscape	2	0.01	0.01	0.61	0.55
Scenario: Landscape	4	0.15	0.04	3.35	0.01 *
Residuals	171	1.90	0.01		

Notes: *** *p* < 0.001, * *p* < 0.05, ^•^ *p* < 0.1; only significant results are displayed.

## Data Availability

The data presented in this study are available in insert article and Appendix A here.

## Appendix A

The goodness of fit, specificity, and sensitivity of different classifiers were calculated for different landscape types and different scenarios to help with choosing the most appropriate classifier in this study.

**Table A1:** The goodness of fit, specificity, and sensitivity of different classifiers for different landscape types.

Classifier	Variable (Scenario)		Forest			Desert			Water	
		Goodness of Fit	Sensitivity	Specificity	Goodness of Fit	Sensitivity	Specificity	Goodness of Fit	Sensitivity	Specificity
Alpha_SVM		0.842			0.838			0.597		
SVM	Landscape		0.958	0.988		0.942	0.946		0.825	0.971
SVM	Structure		1.000	0.954		0.950	0.971		1.000	0.992
SVM	Color		0.892	0.983		0.942	1.000		0.933	0.917
Beta_SVM		0.952			0.770			0.780		
SVM	Landscape		1.000	1.000		0.983	0.946		1.000	0.942
SVM	Structure		1.000	0.950		1.000	1.000		0.950	1.000
SVM	Color		0.900	1.000		0.892	0.992		0.892	0.979
Gamma_SVM		0.944			0.912			0.971		
SVM	Landscape		1.000	0.975		0.992	0.975		0.992	0.988
SVM	Structure		0.942	0.971		0.983	1.000		0.967	0.988
SVM	Color		0.942	0.996		0.967	0.996		0.983	0.996
Alpha_RF		0.365			0.672			0.481		
RF	Landscape		0.800	0.925		0.817	0.883		0.875	0.917
RF	Structure		0.967	0.917		0.858	0.896		0.975	0.983
RF	Color		0.700	0.892		0.883	1.000		0.800	0.925
Beta_RF		0.938			0.639			0.778		
RF	Landscape		0.967	0.992		0.925	0.933		0.983	0.942
RF	Structure		1.000	0.971		0.992	1.000		0.925	0.979
RF	Color		0.958	1.000		0.875	0.963		0.883	0.975
Gamma_RF		0.924			0.787			0.824		
RF	Landscape		0.983	0.975		1.000	0.942		0.992	0.921
RF	Structure		0.917	0.996		1.000	1.000		0.875	0.983
RF	Color		0.992	0.975		0.883	1.000		0.917	0.988
Alpha_KNN		0.430			0.437			0.468		
KNN	Landscape		0.867	0.850		0.650	0.858		0.758	0.938
KNN	Structure		0.917	0.929		0.800	0.829		0.900	0.958
KNN	Color		0.700	0.963		0.817	0.946		0.883	0.875
Beta_KNN		0.655			0.744			0.823		
KNN	Landscape		0.833	0.967		0.975	0.929		0.983	0.963
KNN	Structure		1.000	0.971		0.992	0.988		0.992	1.000
KNN	Color		0.933	0.946		0.867	1.000		0.925	0.988
Gamma_KNN		0.819			0.708			0.835		
KNN	Landscape		0.950	0.950		1.000	0.917		0.958	0.971
KNN	Structure		0.942	1.000		1.000	1.000		0.950	0.971
KNN	Color		0.958	0.975		0.833	1.000		0.925	0.975

**Table A2:** The goodness of fit, specificity, and sensitivity of different classifiers for different scenarios.

Classifier	Variable (Landscape Type)	Landscape Images			Structure Images			Color Images		
		Goodness of Fit	Sensitivity	Specificity	Goodness of Fit	Sensitivity	Specificity	Goodness of Fit	Sensitivity	Specificity
Alpha_SVM		0.642			0.331			0.780		
SVM	Forest		0.842	0.938		0.783	0.892		0.950	0.992
SVM	Desert		0.975	0.954		0.842	0.950		0.892	0.938
SVM	Water		0.900	0.967		0.750	0.846		0.867	0.925
Beta_SVM		0.825			0.911			0.883		
SVM	Forest		0.942	0.983		0.933	0.988		1.000	0.988
SVM	Desert		1.000	1.000		0.933	0.958		0.950	0.954
SVM	Water		0.967	0.971		0.975	0.975		0.883	0.975
Gamma_SVM		0.960			0.901			0.920		
SVM	Forest		1.000	0.975		0.942	0.979		1.000	0.992
SVM	Desert		0.958	0.996		0.950	0.925		1.000	0.950
SVM	Water		0.983	1.000		0.908	0.996		0.883	1.000
Alpha_RF		0.460			0.281			0.460		
RF	Forest		0.725	0.850		0.817	0.825		0.758	0.950
RF	Desert		0.817	0.913		0.800	0.863		0.733	0.888
RF	Water		0.867	0.942		0.558	0.900		0.808	0.813
Beta_RF		0.663			0.653			0.617		
RF	Forest		0.800	0.983		0.950	0.929		0.933	0.925
RF	Desert		0.958	0.971		0.892	0.933		0.858	0.938
RF	Water		0.975	0.913		0.783	0.950		0.792	0.929
Gamma_RF		0.931			0.946			0.874		
RF	Forest		0.992	0.988		0.958	1.000		1.000	0.971
RF	Desert		0.958	1.000		1.000	0.963		0.967	0.967
RF	Water		0.983	0.979		0.958	0.996		0.892	0.992
Alpha_KNN		0.208			0.086			0.384		
KNN	Forest		0.667	0.713		0.725	0.771		0.650	0.888
KNN	Desert		0.717	0.867		0.733	0.904		0.583	0.900
KNN	Water		0.650	0.938		0.475	0.792		0.892	0.775
Beta_KNN		0.320			0.534			0.541		
KNN	Forest		0.575	0.942		0.858	0.900		0.833	0.925
KNN	Desert		0.967	0.950		0.933	0.975		0.900	0.967
KNN	Water		0.917	0.838		0.850	0.946		0.875	0.913
Gamma_KNN		0.592			0.879			0.720		
KNN	Forest		0.775	0.992		0.942	0.979		0.925	0.888
KNN	Desert		1.000	1.000		0.950	0.967		0.825	0.954
KNN	Water		0.983	0.888		0.958	0.979		0.892	0.979
